# The Prevalence of Obesity and Metabolic Syndrome among Polish Women without Pre-Existing Cardiovascular Conditions and Diabetes: A Multicenter Study in Poland

**DOI:** 10.3390/jcm13175014

**Published:** 2024-08-24

**Authors:** Mateusz Babicki

**Affiliations:** Department of Family Medicine, Faculty of Medicine, Wroclaw Medical University, 50-367 Wrocław, Poland; ma.babicki@gmail.com

**Keywords:** HLCPQ, metabolic syndrome, obesity, lifestyle

## Abstract

**Background:** A very prevalent problem worldwide is not only the high prevalence of chronic conditions but also the high frequency of their underdiagnosis and unhealthy lifestyles, both resulting in the development and inadequate treatment of civilization diseases. Therefore, the aim of this study was to assess the prevalence of abnormal metabolic parameters among Polish women aged >35 years who have not yet been diagnosed with cardiovascular diseases, diabetes or chronic kidney disease, as well as evaluate their lifestyles and look for relationships between individual lifestyle parameters and metabolic abnormalities. **Methods**: This was a multicenter, cross-sectional, observational study conducted on a group of women aged ≥ 35 years without previous cardiovascular disease, diabetes or chronic kidney disease. As part of the study, patients had anthropometric measurements and laboratory tests performed (serum glucose, total cholesterol, LDL, HDL, non-HDL cholesterol and triglycerides) and completed the HLPCQ (the Healthy Lifestyle and Personal Control Questionnaire). Obesity was defined as BMI ≥ 30 kg/m^2^. Abdominal obesity was defined as a waist circumference ≥ 88 cm. **Results**: The study included 672 women considered healthy. In the analyzed group of women, 20.6% met the criteria for a diagnosis of obesity based on BMI, and 36.8% were diagnosed with abdominal obesity. In addition, 16.8% of the women had an abnormal fasting blood glucose result. Moreover, 46.4% of the women’s blood pressure measurements were above the normal range, and as many as 57.7% of the women had abnormal non-HDL levels. In addition, 150 women met the criteria for a diagnosis of metabolic syndrome. These conditions were far more common in women diagnosed with obesity. Physical activity was associated with a reduced risk of developing obesity and metabolic syndrome. **Conclusions**: The underdiagnosis of chronic conditions in the study population is high. More than 20% of women met the criteria for a diagnosis of metabolic syndrome, the prevalence of which was significantly higher in patients with obesity. A healthy lifestyle was associated with a reduced risk of developing metabolic syndrome and its individual components. It is necessary to actively search for chronic conditions in patients, which requires the involvement of not only healthcare system employees but also government representatives.

## 1. Introduction

Cardiovascular diseases, diabetes and obesity are now included among the so-called diseases of civilization, the prevalence of which is increasing every year [1]. A huge problem is that some of the diseases of civilization, e.g., chronic kidney disease, type 2 diabetes and hypertension, may go undiagnosed for a long time [2,3,4]. This leads to a lack of appropriate treatment and thus a deteriorating quality of life for patients and can have an adverse effect on their healthy life expectancy. In addition, these conditions can lead to emerging complications, such as diabetic kidney disease, polyneuropathy or myocardial damage, even at an early stage [5]. For example, according to 2019 data from the International Diabetes Federation (IDF), the prevalence of diabetes is 9.3%. The IDF also estimates that, in Europe, one in three patients (36%) living with diabetes is not properly diagnosed, while in Southeast Asia, up to one in two patients with diabetes is not diagnosed [3]. This is supported by data from Thailand, among others, where the prevalence of diabetes was found to be 9.5%, and 30.6% of patients with diabetes were not properly diagnosed [6]. For hypertension, estimates are even less optimistic. It is estimated that hypertension affects more than 1.26 billion people worldwide, of whom as many as 46% may be unaware of their disease. Proper treatment is also another problem, as it is estimated that as many as one in five patients may not be receiving treatment [7]. A study of more than 126,000 patients between the ages of 19 and 64 found that as many as 37.3% had undiagnosed hypertension, and 27% of the percentage of patients with previously diagnosed hypertension did not follow medical advice [2]. The percentages of undiagnosed hypertension in men and women are similar, but with a slight advantage for men [8,9,10].

It is also important to consider obesity, which, firstly, is becoming a growing health problem for people around the world and, secondly, if left untreated, can lead to up to 200 health complications. According to the most recent data, one in eight people worldwide may suffer from obesity [11]. In Europe, it is estimated that about 20% of people are living with obesity [12]. At the same time, depending on the region of the world, the disproportion between women and men with obesity may vary. Some data show a greater prevalence of obesity in women, some data indicate that it is more common in men, and some show no differences [13,14]. Polish data show that up to 18% of women may suffer from obesity [15]. Many of these conditions coexist together; moreover, there is a bidirectional relationship. For example, obesity can cause diabetes, while diabetes increases the risk of developing obesity. Nowadays, the term “diabesity” is beginning to appear in the literature, indicating a close relationship between diabetes and obesity. Obesity also increases the risk of developing cardiovascular disease, dyslipidemia and even liver disease [16].

Such a significant prevalence and underdiagnosis of chronic conditions have a huge impact on both the health of the individual and the burden on healthcare systems, as well as a significant financial burden on state budgets [5]. According to the World Health Organization (WHO), more than 17 million worldwide die annually from non-communicable diseases before the age of 70, making them premature deaths, among which more than 80% are due to cardiovascular diseases, cancer, diabetes or chronic respiratory diseases. The priority to reduce the number of premature deaths undoubtedly requires actively looking for diseases, especially in at-risk groups, and diagnosing them early, which also enables early and correct treatment. Moreover, a key element, both preventive and curative, is proper lifestyle education, which has a tremendous impact on our health [4]. For example, it is currently estimated that smoking is responsible for more than 8 million deaths a year, and more than 830,000 deaths can be attributed to insufficient physical activity. Metabolic factors include overweight/obesity, elevated blood pressure, hyperglycemia and hyperlipidemia, among which, globally, hypertension is a major factor, accounting for up to 19% of deaths worldwide [17]. It is worth noting that all of these metabolic factors blend into the definition of metabolic syndrome, which includes the presence of obesity or abdominal obesity and the presence of at least two of three factors: elevated blood pressure, impaired glucose metabolism or elevated serum non-HDL cholesterol [18]. For example, in 2014, the prevalence of metabolic syndrome in Poland was 33% among women, and this percentage is likely to increase with each passing year [19]. One of the therapeutic as well as preventive approaches is lifestyle modification, including regular physical activity, the modification of eating habits and smoking cessation [20].

Lifestyle is an important modifiable factor affecting the risk of developing many chronic conditions and life expectancy. Moreover, it is also believed that a healthy lifestyle extends our life expectancy without chronic conditions. This has been confirmed in a group of more than 116,000 patients [21]. Therefore, the aim of this study was to assess the prevalence of abnormal metabolic parameters among Polish women aged > 35 years who have not yet been diagnosed with cardiovascular diseases, diabetes or chronic kidney disease, as well as assess their lifestyles and search for relationships between individual lifestyle parameters and metabolic abnormalities.

## 2. Materials and Methods

This was a multicenter, cross-sectional, observational study of women aged ≥ 35 years without a prior diagnosis of cardiovascular disease, diabetes or chronic kidney disease. Inclusion criteria for the study included the following:

(a) Being a woman;
(b) Age ≥ 35 years;
(c) No previously diagnosed cardiovascular conditions, diabetes or chronic kidney disease;
(d) Informed, written consent to participate in the study;
(e) Performance of laboratory tests within the past few weeks, which included a lipid panel (total cholesterol, HDL, LDL, non-HDL, triglycerides) and fasting serum glucose levels;
(f) The completion of the HLPCQ (the Healthy Lifestyle and Personal Control Questionnaire).

For inclusion in the study, it was necessary to meet all inclusion criteria. If even 1 of the above was not met, the patient was not included in the analysis. There were 10 primary care facilities involved in the recruitment process and the data collection. Randomized women who met the inclusion criteria at the time of the visit were included in the study.

Patients had a primary care medical visit, which included a physical examination, an analysis of test results and anthropometric measurements.

During the visit, a history was collected regarding age, gender, place of residence and education level. Questions were also asked about the family burden of cardiovascular diseases—the occurrence of stroke and/or myocardial infarction in the mother and/or father. In addition, cigarette smoking status was assessed with details on whether they smoked currently, smoked in the past, passively smoked or never smoked. Physical activity was also surveyed. Moderate activity was defined as activity that causes faster breathing and a faster heart rate (e.g., cycling at a normal pace, playing volleyball or taking a brisk walk). In contrast, intense physical activity was defined as activity that causes very fast breathing and a very fast heart rate (e.g., lifting weights, aerobic exercise, running, cycling at a faster pace). Both the frequency of activity (none at all, 1–2 times a week, 3–4 times a week, >4 times a week) and the average number of minutes per week were assessed. In the next step, patients had anthropometric measurements taken (height, weight, waist circumference). Waist circumference was measured according to current recommendations, i.e., at a point halfway between the ribcage arch and the highest point of the iliac crest in the median axillary line [18]. BMI was calculated based on weight and height. Patients also had their blood pressure and heart rate measured. Measurements were taken during the medical visit using sphygmomanometers dedicated to adult patients. Each patient had their blood pressure measured three times, from which the average value was determined.

The next stage of the visit included analysis of laboratory results—serum glucose, total cholesterol, LDL, HDL, non-HDL cholesterol and triglycerides.

The final stage of the study involved patients completing the Healthy Lifestyle and Personal Control Questionnaire (HLPCQ). The questionnaire was completed in the doctor’s office, which saved time and increased the women’s response rate on the questionnaires. The above questionnaire was developed in 2001 and was validated for the Polish population in 2021 by Czapla et al. [22] The questionnaire consists of 21 closed questions based on a 4-point Likert scale, where 1—never or rarely; 2—sometimes; 3—often; 4—always. The questions relate to selected lifestyle elements. The maximum number of points that can be scored on the test is 104 points. The analysis of the scale is based on the total score, where the more points, the healthier the lifestyle. In addition to the overall analysis, it is also possible to analyze 5 subscales:

(1) Healthy dietary choices—questions 1, 3, 4, 5, 13, 14 and 16; maximum number of points—28.
(2) Avoidance of harmful diets—questions 8, 9, 10 and 11; maximum number of points—16.
(3) Daily routine—questions 2, 6, 7, 12, 15, 17, 19 and 22; maximum number of points—32.
(4) Organized physical exercise—questions 20 and 23; maximum number of points—8.
(5) Social and mental balance—questions 18, 21, 24, 25 and 26; maximum number of points—20 [22].

The HLPCQ in the above study has a high internal consistency, with a Cronbach’s alpha of 0.887.

The criteria for the diagnosis of metabolic syndrome were based on the 2022 definition proposed by an expert panel. To diagnose metabolic syndrome, a patient must meet one of the following criteria:

(1) Established obesity (defined as BMI ≥ 30 kg/m^2^);
(2) Established abdominal obesity, defined as a waist circumference ≥ 88 cm.

And, at least 2 of the 3 following abnormalities must be present:

(1) Serum fasting glucose ≥ 100 mg/dL;
(2) Serum non-HDL ≥ 130 mg/dL;
(3) Blood pressure ≥ 130 and/or 85 mmHg [18].

This study was conducted in accordance with the Declaration of Helsinki, and the approval of the Bioethics Committee of Wroclaw Medical University was obtained (decision No. KB-540/2022).

### Statistical Analysis

Qualitative and quantitative variables were analyzed. The normality of distributions was assessed using the Shapiro–Wilk test. Comparisons of qualitative variables were made using the Chi-square test. For qualitative variables, the non-parametric Mann–Whitney U test was used. In the next stage, in order to evaluate the influence of lifestyle on the occurrence of health problems, complex logistic regression models were conducted, where the independent variables were age and the total score of the HLPCQ scale and the scores of its individual subscales. The dependent variables included BMI ≥ 30, waist circumference ≥ 88 cm, serum fasting glucose ≥ 100 mg/dL, serum non-HDL ≥ 130 mg/dL, blood pressure ≥ 130 and/or 85 mmHg and the presence of metabolic syndrome. Statistical significance was taken as <0.05. Calculations were performed using Statistical 13 software from TIBCO Software Inc. (Palo Alto, CA, USA).

## 3. Results

### 3.1. Description of the Study Group

This study included 672 women with a mean age of 47.9 ± 9.3 who were previously untreated for cardiovascular conditions and diabetes. The vast majority of the women worked in a white-collar job (47.9%) and lived in a city with >50,000 inhabitants (44.2%). Additionally, 61.9% of the women declared that they do not smoke, but only 59.7% reported regular physical activity at least once a week.

In the study group, 139 (20.6%) women met the criterion for a diagnosis of obesity based on BMI. There were no differences in regular physical activity between women with and without obesity, taking into account both the frequency and duration of activity. Women with higher education were less likely to be diagnosed with obesity compared to other women (*p* = 0.004), as were women with white-collar jobs (*p* < 0.001). A total of 46.8% of women with obesity and 38.6% of women without obesity did not engage in any kind of physical activity.

A detailed summary of the description of the study group, including the division into those with and without obesity, is presented in Table 1.

### 3.2. Laboratory Test Results

It was shown that women diagnosed with obesity showed significantly worse laboratory results than women without obesity. They were characterized by higher blood pressure values (both systolic and diastolic) and lipid disorders (higher concentrations of total cholesterol, LDL and non-HDL). There were no differences in heart rate.

A comparison of the test results of the women studied is shown in Table 2.

### 3.3. Health Complications in Potentially Healthy Women

In the analyzed group of women who did not have any diagnosed chronic conditions prior to their visit to the doctor, 20.6% met the criteria for a diagnosis of obesity based on their BMI, and 36.8% were diagnosed with abdominal obesity. In addition, 16.8% of the women had an abnormal fasting blood glucose result. Moreover, 46.4% of the women’s blood pressure measurements were above the normal range, and as many as 57.7% of the women had abnormal non-HDL levels. In addition, 150 women met the criteria for a diagnosis of metabolic syndrome.

The aforementioned conditions were far more common in obese patients. In this group of patients, as many as 74.1% had abnormal non-HDL values, 72.7% of patients obtained abnormal values of blood pressure measurements, and 33.8% had glycemic values ≥ 100 mg/dL. A detailed summary is shown in Table 3.

### 3.4. Lifestyle

The analysis of HLPCQ scale scores showed that obese patients scored significantly lower on subscales relating to daily routine (*p* = 0.004) and exercise (*p* = 0.005). In addition, obese patients scored overall 3.3 points lower (*p* = 0.007). A detailed comparison is shown in Table 4.

### 3.5. Effect of Lifestyle on Health Problems

Multivariate logistic regression analysis showed that a healthy lifestyle was associated with a lower risk of elevated non-HDL cholesterol (OR= 0.97) and a reduced risk of developing BMI-defined obesity (OR = 0.97) and abdominal obesity (OR = 0.98). In addition, regular physical activity also contributes to reducing the development of obesity. A detailed summary of the results of multivariate logistic regression is presented in Table 5.

## 4. Discussion

The aim of the above study was to assess the prevalence of abnormal metabolic parameters among Polish women aged ≥ 35 years who have not yet been diagnosed with cardiovascular diseases, diabetes or chronic kidney disease, as well as to assess their lifestyles and to look for relationships between individual lifestyle parameters and metabolic abnormalities. For this purpose, 672 women considered healthy were included in the study. In the study group, a significant percentage of patients were found to have metabolic abnormalities. For example, 20.6% of the women met the criteria for a diagnosis of obesity based on BMI and 36.8% for abdominal obesity based on waist circumference measurement. These data are consistent with previous observations, where the prevalence of obesity among women in Poland was estimated at about 18% [15]. The prevalence of obesity varies by region of the world, affecting both developed and developing countries [1,12,17,23]. Interestingly, it is believed that women are almost twice as likely as men to develop obesity. The etiopathogenesis of this phenomenon is complex, but the role of genetic factors, the history of pregnancy, the use of hormonal contraception, hormonal differences (e.g., insulin/insulin-like growth factor ratio, sex hormones) and the important role of adipocytokines contribute [24,25,26].

Obesity is associated with many health complications. It is currently estimated that it can cause up to 200 complications, including an increased risk of developing cardiovascular disease, dyslipidemia, type 2 diabetes, psychiatric conditions and degenerative joint changes [27]. These complications affect both sexes. Nevertheless, the literature data suggest that some women may be more predisposed to their development. For example, a large meta-analysis showed that the effect of BMI on the risk of developing type II diabetes is significantly higher in women than in men [26]. Our study showed that women with obesity had higher mean fasting blood glucose. In addition, as many as one in three obese patients had a glucose result > 100 mg/dL, which equates to a further diagnosis of diabetes.

Our study also showed that patients with obesity were significantly more likely to have abnormal blood pressure values (72.7% vs. 39.6%, *p* < 0.001), which is in line with scientific evidence that up to 65% of primary hypertension in women may be just induced by obesity [28]. The potential mechanisms by which obesity causes hypertension are complex and include the excessive activation of the sympathetic nervous system, the stimulation of the renin–angiotensin–aldosterone system, changes in adipose tissue-derived cytokines, insulin resistance and structural and functional changes in the kidneys [29]. Finally, the values of total cholesterol, LDL, non-HDL cholesterol and triglycerides were higher in patients with obesity-related diseases than in those with normal weight. These observations are consistent with scientific evidence, where it has repeatedly been shown that patients with obesity-related diseases are characterized by abnormal lipidograms, especially high levels of triglycerides, non-HDL and apolipoprotein B. It is estimated that this problem can affect up to 60–70% of patients with obesity and 50–60% of overweight patients. Children with obesity can also be affected [30].

What should be emphasized is that the analyzed group consisted of women who had not previously been diagnosed with chronic conditions, among whom a significant percentage of undiagnosed conditions were found. In the entire study group, 16.8% had blood glucose levels above 100 mg/dL, 57.7% had excessively high non-HDL cholesterol levels, 46.4% had excessively high blood pressure values, and 20.6% met the criteria for a diagnosis of obesity. Moreover, one in five women surveyed (22.3%) met the criteria for a diagnosis of metabolic syndrome. These results clearly indicate that the problem of undiagnosed chronic conditions in such groups of women is widespread, and the results obtained are consistent with international data. A publication by showed that undiagnosed hypertension was present in England about 27.6% [9]. The same is true for diabetes, where, based on data from 748,046 patients, undiagnosed diabetes was shown to occur in nearly 24.2 percent of women [31]. The same is true for metabolic syndrome, which is more common in women, and most people may be unaware of their disease [32,33].

Failure to diagnose a disease leads to a lack of appropriate treatment, which ultimately contributes to worsening health, worsening life expectancy, and further health complications. Many of these conditions are associated with inappropriate lifestyles, including physical inactivity, smoking or poor eating habits [2,4,11,15,16,17,18,34]. This is also supported by our results, where measured lifestyle parameters using the HLPCQ were associated with a reduced risk of developing metabolic syndrome, abnormal cholesterol levels or obesity. In complex logistic regression models, regular physical activity was shown to reduce the risk of developing obesity, abdominal obesity and metabolic syndrome. Regular physical activity is a recognized factor in reducing the risk of developing metabolic syndrome and its individual components, as well as overall mortality. Even moderate physical activity has been shown to improve insulin sensitivity, lipid profile and blood pressure [34]. In addition, the anti-inflammatory effects of physical activity help reduce oxidative stress and decrease the release of pro-inflammatory adipokines. And, finally, regular physical activity contributes to increased calories burned, thereby improving energy expenditure. An inverse relationship has been shown between regular physical activity and the amount of visceral fat. This is also supported by our results, where higher values of the “Organized physical exercise” subscale were associated with a lower risk of developing abdominal obesity [34,35].

Thus, a healthy lifestyle is a key element in both the prevention of chronic conditions and their appropriate treatment. It would seem to represent one of the lowest-cost and most effective forms of treatment for conditions such as metabolic syndrome. This research shows that adherence to medical recommendations for lifestyle changes is very low. Patients are reluctant to implement regular physical activity or modify their eating habits [36].

Analyzing the results of the above study, where a high percentage of undiagnosed civilization diseases and the positive impact of a healthy lifestyle were shown, it would be advisable to strive for the increased implementation of preventive examinations in groups of patients with risk factors and to place more emphasis on appropriate education on healthy lifestyles.

Opportunities for government involvement in improving the diagnosis of civilization diseases in Poland undoubtedly include encouraging both patients and primary care units to implement preventive programs. In addition, it is necessary to constantly conduct educational campaigns aimed at patients to raise their awareness of chronic conditions and the complications of diseases such as obesity. For this purpose, it is possible to use mass media, such as Television, the Internet or radio. One solution could also be to send out invitations to realize prevention programs. The authors are aware of the limitations of the above study, which is undoubtedly the lack of representativeness of the analyzed group for the Polish female population. In addition, we do not have data on the use of medications and supplements (other than those used in the treatment of cardiovascular diseases, diabetes and chronic kidney disease), which could potentially affect laboratory results and patients’ weight. Another limitation relates to the lifestyle elements analyzed, without including variables such as sleep, alcohol consumption and other stimulants. We should also mention the limitation of the study due to the lack of analysis of renal parameters.

In summary, the above work provides data showing that the prevalence of undiagnosed metabolic conditions in the study population is high. One in five women surveyed suffered from metabolic syndrome, more than half had abnormal non-HDL cholesterol values, and 46.4% obtained abnormal blood pressure measurements. Moreover, these conditions are far more common in obese women. It has also been shown that a healthy lifestyle, especially regular physical activity, undoubtedly contributes to reducing the risk of developing obesity and metabolic syndrome. The high percentage of undiagnosed health problems among a potentially healthy population of Polish women indicates how important it is to actively seek out chronic diseases in patients. It also shows how important a role the family doctor plays in the whole process at the stages of both diagnosis and treatment. In addition, a key role of the family physician should be the promotion of healthy lifestyles and preventive measures. Moreover, in order to significantly improve the diagnosis of chronic diseases and their treatment, it is necessary to have greater involvement of healthcare system professionals but also governments, which, through top-down actions, should encourage people to, among other things, participate in preventive programs, which are undoubtedly an important element of healthcare systems.

## Figures and Tables

**Table 1:** Characteristics of the study group, including the presence of obesity.

Variable		The Whole Group (N = 672)	Without Obesity (N = 533)	Obesity (N = 139)	*p*
Age M ± SD		47.9 ± 9.3	47.1 ± 9.1	51.2 ± 9.3	**<0.001 ^†^**
Weight [kg] M ± SD		70.4 ± 15.3	64.7 ± 8.8	92.3 ± 15.1	**<0.001 ^†^**
Height [cm] M ± SD		164.1 ± 6.4	164.3 ± 6.4	163.2 ± 6.5	0.091 ^†^
Waist circumference [cm] M ± SD		84.5 ± 13.1	79.9 ± 9.1	102.2 ± 10.8	**<0.001 ^†^**
Type of profession	White-collar worker	322 (47.9)	280 (87.0)	42 (13.0)	**<0.001**
	Manual worker	145 (21.6)	114 (78.6)	31 (21.4)	
	Farmer	49 (7.3)	34 (69.4)	15 (30.6)	
	Pensioner	80 (11.9)	45 (65.0)	28 (35.0)	
	Other	76 (11.3)	53 (69.7)	23 (30.3)	
Education	Basic vocational	145 (21.5)	98 (67.5)	47 (23.5)	**0.004**
	Secondary	197 (29.4)	151 (76.7)	46 (23.3)	
	Higher	318 (47.3)	275 (86.4)	43 (13.6)	
	Other	12 (1.2)	9 (75.0)	3 (25.0)	
Place of residence	Village	206 (30.7)	152 (73.8)	54 (26.2)	**0.002**
	City of up to 50,000 inhabitants	169 (25.1)	127 (75.2)	42 (24.8)	
	City of over 50,000 inhabitants	297 (44.2)	254 (85.5)	43 (14.5)	
Heart attack and/or stroke in a father under 55?		57 (8.5)	44 (77.2)	13 (22.8)	0.679 ^‡^
Heart attack and/or stroke in a mother under 65?		29 (4.3)	23 (79.3)	6 (20.7)	0.994 ^‡^
Smoking	Smokes cigarettes	147 (21.9)	110 (74.8)	37 (25.2)	0.408 ^‡^
	Smoked in the past	94 (14.0)	77 (81.9)	17 (18.1)	
	Passively smokes	15 (2.2)	11 (73.3)	4 (26.7)	
	Does not smoke	416 (61.9)	335 (80.5)	81 (19.5)	
Moderate physical activity	I don’t do that kind of activity	271 (40.3)	206 (76.0)	65 (24.0)	0.295 ^‡^
	1–2 times a week	146 (21.7)	122 (83.6)	24 (16.4)	
	3–4 times a week	123 (18.3)	100 (81.3)	23 (18.7)	
	>4 times a week	132 (19.6)	105 (79.6)	27 (20.5)	
Average time of moderate physical activity [min/week]		162.9 ± 374.7	154.3 ± 338.3	196.1 ± 489.9	0.411 ^†^
Intense physical activity	I don’t do that kind of activity	546 (81.3)	430 (78.8)	116 (21.2)	0.614 ^‡^
	1–2 times a week	57 (8.5)	48 (84.2)	9 (15.8)	
	3–4 times a week	42 (6.2)	35 (83.3)	7 (16.7)	
	>4 times a week	27 (4.0)	20 (74.1)	7 (25.9)	
Average time of intense physical activity [min/week]		26.8 ± 96.2	28.7 ± 102.7	19.8 ± 64.9	0.418 ^†^

*p*—statistical significance; M—mean; SD—standard deviation; N—number; ^†^ Mann–Whitney U test; ^‡^ Chi-squared test. Statistically significant values are in bold, with the significance level set at *p* < 0.05.

**Table 2:** A comparison of laboratory results and blood pressure and heart rate measurements for the entire group and in relation to obesity.

Variable	The Whole Group (N = 672)	Without Obesity (N = 533)	Obesity (N = 139)	*p*
SBP [mmHg] M ± SD	126.8 ± 16.9	124.3 ± 16.2	136.5 ± 16.4	<0.001
DBP [mmHg] M ± SD	80.4 ± 9.8	79.1 ± 9.6	84.9 ± 9.2	<0.001
Heart action [beats per minute] M ± SD	75.1 ± 10.7	74.9 ± 10.5	76.0 ± 11.5	0.403
Total cholesterol [mg/dL] M ± SD	205.0 ± 40.8	203.5 ± 41.2	210.8 ± 38.7	0.019
LDL [mg/dL] M ± SD	122.7 ± 38.0	120.8 ± 37.8	129.7 ± 37.9	0.004
HDL [mg/dL] M ± SD	62.9 ± 15.7	64.8 ± 15.4	55.4 ± 14.4	<0.001
Non-HDL [mg/dL] M ± SD	142.5 ± 41.6	138.9 ± 41.4	156.2 ± 39.5	<0.001
Triglycerides [mg/dL] M ± SD	108.0 ± 60.3	99.7 ± 55.3	139.7 ± 67.9	<0.001
Glucose [mg/dL] M ± SD	92.1 ± 16.0	90.3 ± 10.4	99.2 ± 27.7	<0.001

M—mean; SD—standard deviation; *p*—statistical significance; N—number; mmHg—millimeters of mercury; mg/dL—milligrams per deciliter; SBP—Systolic Blood Pressure; DBP—Diastolic Blood Pressure, LDL—low-density lipoprotein; HDL—high-density lipoprotein.

**Table 3:** The prevalence of abnormal results of biochemical tests, anthropometric measurements and metabolic syndrome in the analyzed group, including patients with and without obesity.

Variable	The Whole Group (N = 672)	Without Obesity (N = 533)	Obesity (N = 139)	*p*
Serum fasting glucose ≥ 100 mg/dL	113 (16.8)	66 (12.4)	47 (33.8)	<0.001
Serum non-HDL ≥ 130 mg/dL	388 (57.7)	285 (53.5)	103 (74.1)	<0.001
Blood pressure ≥ 130 and/or 85 mmHg	312 (46.4)	211 (39.6)	101 (72.7)	<0.001
BMI ≥ 30	139 (20.6)	0 (0.0	139 (100.0)	<0.001
Abdominal obesity (waist circumference ≥ 88 cm)	247 (36.8)	115 (21.6)	132 (53.4)	<0.001
Metabolic syndrome	150 (22.3)	60 (40.0)	90 (60.0)	<0.001

**Table 4:** A comparison of HLPCQ scale scores for the whole group and groups with and without the presence of obesity.

HLPCQ	The Whole Group (N = 672)	Without Obesity (N = 533)	Obesity (N = 139)	*p*
Healthy dietary choices	18.0 ± 3.6	18.1 ± 3.6	17.6 ± 3.5	0.146
Dietary harm avoidance	11.3 ± 2.8	11.4 ± 2.8	10.9 ± 3.1	0.096
Daily routine	20.9 ± 5.4	21.2 ± 5.4	19.8 ± 5.5	0.004
Organized physical exercise	4.3 ± 1.8	4.3 ± 1.8	3.9 ± 1.7	0.005
Social and mental balance	13.1 ± 3.0	13.2 ± 3.0	12.9 ± 3.0	0.190
Final result	67.6 ± 12.4	68.3 ± 12.4	65.0 ± 12.2	0.007

**Table 5:** The results of multivariate logistic regression analysis evaluating the effects of lifestyle on abnormal biochemical parameters, obesity and metabolic syndrome among women.

Variable	HLPCQ											
	Healthy Dietary Choices		Dietary Harm Avoidance		Daily Routine		Organized Physical Exercise		Social and Mental Balance		Final Result	
	OR [95 Cl]	*p*	OR [95 Cl]	*p*	OR [95 Cl]	*p*	OR [95 Cl]	*p*	OR [95 Cl]	*p*	OR [95 Cl]	*p*
Serum fasting glucose ≥ 100 mg/dL	0.98 [0.91, 1.06]	0.534	1.02 [0.94, 1.11]	0.662	0.97 [0.93, 1.02]	0.273	0.87 [0.76, 1.01]	0.073	0.98 [0.96, 0.99]	0.048	1.00 [0.98, 1.02]	0.663
Serum non-HDL ≥ 130 mg/dL	0.95 [0.89, 1.01]	0.118	1.02 [0.95, 1.09]	0.622	0.97 [0.93, 1.01]	0.124	0.95 [0.86, 1.06]	0.378	1.01 [0.95, 1.08]	0.633	0.97 [0.95, 0.99]	0.014
Blood pressure ≥ 130 and/or 85 mmHg	0.98 [0.92, 1.04]	0.454	0.99 [0.93, 1.06]	0.775	1.01 [0.98, 1.05	0.456	0.94 [0.84, 1.04]	0.231	1.01 [0.95, 1.08]	0.718	0.95 [0.92, 1.05]	0.213
BMI ≥ 30	1.03 [0.96, 1.12]	0.321	0.94 [0.87, 1.01]	0.113	0.94 [0.91, 0.99]	0.017	0.88 [0.77, 0.98]	0.045	1.03 [0.95, 1.11]	0.407	0.97 [0.96, 0.99]	0.007
Abdominal obesity (waist circumference ≥ 88 cm)	1.01 [0.95, 1.07]	0.741	0.93 [0.87, 0.99]	0.032	0.97 [0.93, 1.00]	0.064	0.88 [0.79, 0.99]	0.034	1.05 [0.98, 1.13]	0.112	0.98 [0.96, 0.99]	0.004
Metabolic syndrome	0.97 [0.89, 1.03]	0.343	0.99 [0.92, 1.07]	0.822	0.97 [0.93, 1.02]	0.216	0.88 [0.77, 0.98]	0.048	1.07 [0.99, 1.17]	0.073	0.98 [0.97, 1.00]	0.171

## Data Availability

The data presented in this study are available on request from the corresponding author.

## References

[1] Clatici V.G., Voicu C., Voaides C., Roseanu A., Icriverzi M., Jurcoane S. (2018). Diseases of Civilization—Cancer, Diabetes, Obesity and Acne—The Implication of Milk, IGF-1 and MTORC1. Maedica.

[2] Huguet N., Larson A., Angier H., Marino M., Green B.B., Moreno L., Devoe J.E. (2021). Rates of Undiagnosed Hypertension and Diagnosed Hypertension without Anti-Hypertensive Medication Following the Affordable Care Act. Am. J. Hypertens..

[3] IDF IDF Diabetes Atlas. 2021. IDF Off. Website 2021. pp. 1–4. https://diabetesatlas.org/.

[4] Singer M. (2014). Non-Communicable Diseases. The Wiley Blackwell Encyclopedia of Health, Illness, Behavior, and Society.

[5] Gambert S.R. (2006). The Burden of Chronic Disease. Clin. Geriatr..

[6] Aekplakorn W. The Thai National Health Examination Survey. https://assets.adobe.com/public/339f7919-70a0-4d09-4a3a-41e4c74e3965.

[7] Hypertension. https://www.who.int/news-room/fact-sheets/detail/hypertension.

[8] Uhernik A.I., Kralj V., Čukelj P., Brkić-Biloš I., Erceg M., Benjak T., Stevanović R. (2023). Undiagnosed Hypertension in Croatia. Croat. Med. J..

[9] Campbell E., Macey E., Shine C., Nafilyan V., Clark N.C., Pawelek P., Ward I., Hughes A., Raleigh V., Banerjee A. (2023). Sociodemographic and Health-Related Differences in Undiagnosed Hypertension in the Health Survey for England 2015–2019: A Cross-Sectional Cohort Study. eClinicalMedicine.

[10] Chau K., Girerd N., Zannad F., Rossignol P., Boivin J.M. (2018). Health-Related Determinants of Undiagnosed Arterial Hypertension: A Population-Based Study. Fam. Pract..

[11] Vaamonde J.G., Álvarez-Món M.A. (2020). Obesity and Overweight. Medicine.

[12] Eurostat (2021). Overweight and Obesity—BMI Statistics—Statistics Explained. Eurostat Stat. Explain..

[13] Boutari C., Mantzoros C.S. (2022). A 2022 Update on the Epidemiology of Obesity and a Call to Action: As Its Twin COVID-19 Pandemic Appears to Be Receding, the Obesity and Dysmetabolism Pandemic Continues to Rage On. Metabolism.

[14] Stierman B., Afful J., Carroll M.D., Chen T.C., Davy O., Fink S., Fryar C.D., Gu Q., Hales C.M., Hughes J.P. (2021). National Health and Nutrition Examination Survey 2017–March 2020 Prepandemic Data Files Development of Files and Prevalence Estimates for Selected Health Outcomes. Natl. Health Stat. Rep..

[15] Profilaktyka i Leczenie Otyłości Przerosły System (Zapis Konferencji Prasowej)—Najwyższa Izba Kontroli. https://www.nik.gov.pl/aktualnosci/coraz-wiecej-osob-z-choroba-otylosciowa-coraz-dluzsze-kolejki.html.

[16] Chandrasekaran P., Weiskirchen R. (2024). The Role of Obesity in Type 2 Diabetes Mellitus—An Overview. Int. J. Mol. Sci..

[17] Mathers C. (2016). Global Burden of Disease. International Encyclopedia of Public Health.

[18] Dobrowolski P., Prejbisz A., Kuryłowicz A., Baska A., Burchardt P., Chlebus K., Dzida G., Jankowski P., Jaroszewicz J., Jaworski P. (2022). Metabolic Syndrome—A New Definition and Management Guidelines. Arter. Hypertens..

[19] Rajca A., Wojciechowska A., Śmigielski W., Drygas W., Piwońska A., Pająk A., Tykarski A., Kozakiewicz K., Kwaśniewska M., Zdrojewski T. (2021). Increase in the Prevalence of Metabolic Syndrome in Poland: Comparison of the Results of the WOBASZ (2003–2005) and WOBASZ II (2013–2014) Studies. Pol. Arch. Intern. Med..

[20] Al Thani M., Al Thani A.A., Al-Chetachi W., Al Malki B., Khalifa S.A.H., Bakri A.H., Hwalla N., Nasreddine L., Naja F. (2016). A ‘High Risk’ Lifestyle Pattern Is Associated with Metabolic Syndrome among Qatari Women of Reproductive Age: A Cross-Sectional National Study. Int. J. Mol. Sci..

[21] Nyberg S.T., Singh-Manoux A., Pentti J., Madsen I.E.H., Sabia S., Alfredsson L., Bjorner J.B., Borritz M., Burr H., Goldberg M. (2020). Association of Healthy Lifestyle with Years Lived Without Major Chronic Diseases. JAMA Intern. Med..

[22] Czapla M., Juárez-Vela R., Rozensztrauch A., Karniej P., Uchmanowicz I., Santolalla-Arnedo I., Baska A. (2021). Psychometric Properties and Cultural Adaptation of the Polish Version of the Healthy Lifestyle and Personal Control Questionnaire (Hlpcq). Int. J. Environ. Res. Public Health.

[23] Phelps N.H., Singleton R.K., Zhou B., Heap R.A., Mishra A., Bennett J.E., Paciorek C.J., Lhoste V.P., Carrillo-Larco R.M., Stevens G.A. (2024). Worldwide Trends in Underweight and Obesity from 1990 to 2022: A Pooled Analysis of 3663 Population-Representative Studies with 222 Million Children, Adolescents, and Adults. Lancet.

[24] Escobar-Morreale H.F., Santacruz E., Luque-Ramírez M., Carretero J.I.B. (2017). Prevalence of “obesity-Associated Gonadal Dysfunction” in Severely Obese Men and Women and Its Resolution after Bariatric Surgery: A Systematic Review and Meta-Analysis. Hum. Reprod. Update.

[25] Lewitt M., Dent M., Hall K. (2014). The Insulin-Like Growth Factor System in Obesity, Insulin Resistance and Type 2 Diabetes Mellitus. J. Clin. Med..

[26] Censin J.C., Peters S.A.E., Bovijn J., Ferreira T., Pulit S.L., Mägi R., Mahajan A., Holmes M.V., Lindgren C.M. (2019). Causal Relationships between Obesity and the Leading Causes of Death in Women and Men. PLoS Genet..

[27] MedSci K.K.F., Schnecke V., Haase C.L., Harder-Lauridsen N.M., Rathor N., Sommer K., Morgen C.S. (2023). Weight Change and Risk of Obesity-Related Complications: A Retrospective Population-Based Cohort Study of a UK Primary Care Database. Diabetes Obes. Metab..

[28] Abohelwa M., Kopel J., Shurmur S., Ansari M.M., Awasthi Y., Awasthi S. (2023). The Framingham Study on Cardiovascular Disease Risk and Stress-Defenses: A Historical Review. J. Vasc. Dis..

[29] Shariq O.A., Mckenzie T.J. (2020). Obesity-Related Hypertension: A Review of Pathophysiology, Management, and the Role of Metabolic Surgery. Gland Surg..

[30] Nussbaumerova B., Rosolova H. (2023). Obesity and Dyslipidemia. Curr. Atheroscler. Rep..

[31] Sahadevan P., Kamal V.K., Sasidharan A., Bagepally B.S., Kumari D., Pal A. (2023). Prevalence and Risk Factors Associated with Undiagnosed Diabetes in India: Insights from NFHS-5 National Survey. J. Glob. Health.

[32] Basu S., James Thirunavukarasu A., Maheshwari V., Zode M., Hassan R. (2023). Burden, Determinants and Treatment Status of Metabolic Syndrome among Older Adults in India: A Nationally Representative, Community-Based Cross-Sectional Survey. BMJ Public Health.

[33] Mastwyk S., Taylor N.F., Lowe A., Dalton C., Peiris C.L. (2024). Metabolic Syndrome Is Prevalent and Undiagnosed in Clients Attending Private Practice Physiotherapy: A Cross-Sectional Study. Physiotherapy.

[34] Chomiuk T., Niezgoda N., Mamcarz A., Śliż D. (2024). Physical Activity in Metabolic Syndrome. Front. Physiol..

[35] Niemiro G.M., Rewane A., Algotar A.M. Exercise and Fitness Effect on Obesity. https://pubmed.ncbi.nlm.nih.gov/30969715/.

[36] Andualem A., Gelaye H., Damtie Y. (2020). Adherence to Lifestyle Modifications and Associated Factors among Adult Hypertensive Patients Attending Chronic Follow-up Units of Dessie Referral Hospital, North East Ethiopia, 2020. Integr. Blood Press. Control.

